# Effects of melatonin on muscle and liver bioenergetic profiles: focus on acylcarnitines following endurance swimming exercise in rats

**DOI:** 10.1007/s42000-026-00796-x

**Published:** 2026-06-08

**Authors:** Alinson Eduardo Cipriano, Alex Aparecido Rosini Silva, Vanessa Bertolucci, Andreia de Melo Porcari, Leonardo Henrique Dalcheco Messias, Wladimir Rafael Beck

**Affiliations:** 1 Laboratory of Endocrine Physiology and Physical Exercise, Department of Physiological Sciences, Federal University of São Carlos, São Carlos, 13565-905 Brazil; 2 University of São Caetano do Sul, São Caetano do Sul, 09521-160 Brazil; 3 MS4 Life Laboratory of Mass Spectrometry, Health Sciences Postgraduate Program, São Francisco University, Bragança Paulista, 12916-900 Brazil; 4 Research Group on Technology Applied to Exercise Physiology - GTAFE, Health Sciences Postgraduate Program, São Francisco University, Bragança Paulista, 12916-900 Brazil

**Keywords:** Post-exercise recovery, N-acetyl-5-methoxytryptamine, Metabolism, Lipids, Metabolomics

## Abstract

**Introduction:**

Melatonin (N-acetyl-5-methoxytryptamine) plays a key role in lipid metabolism regulation, directly influencing fatty acid oxidation and lipid transport and modulating pathways related to energy homeostasis.

**Objective:**

This study aimed to evaluate its acute effects on tissue bioenergetics during post-exercise recovery.

**Methods:**

Thirty Wistar rats performed a 60-min swimming at 90% of their individual maximal aerobic capacity and received melatonin (10 mg.kg^-^¹) or vehicle immediately after exercise. The animals were euthanized at 1, 3, or 24 h post-exercise.

**Results:**

Melatonin did not alter the acylcarnitine pool in the muscle or liver, indicating no synergistic effect on β-oxidation. However, it significantly reduced serum glucose levels (F = 11.01; *p* < 0.001), increased glycogen levels in the red gastrocnemius (F = 82.81; *p* < 0.001), gluteus maximus (F = 19.55; *p* < 0.001), and liver (F = 6.24; *p* < 0.001), and decreased triglyceride content in several skeletal muscles. The integrated biomarker response (IBR) revealed time-dependent shifts in specific acylcarnitine species, although melatonin did not influence these shifts.

**Conclusion:**

In conclusion, despite not modulating the acylcarnitine pool, melatonin induced favorable metabolic adaptations by enhancing glycogen replenishment and altering lipid availability in a tissue-specific manner. These findings suggest that melatonin may favor the transport or utilization of larger fatty acid molecules, potentially bypassing the conventional acylcarnitine-mediated oxidation pathways. This highlights its role as a modulator of post-exercise recovery and underscores the need for temporally resolved analyses to unravel its bioenergetic effects.

**Supplementary Information:**

The online version contains supplementary material available at https://doi.org/10.1007/s42000-026-00796-x.

## Introduction

Melatonin, an amphiphilic indoleamine [1], is primarily produced by the pineal gland and plays a key role in the regulation of circadian and seasonal rhythms [2]. It crosses cellular plasma membranes and the blood-brain barrier [3], exerting endocrine action by circulating freely in the plasma or binding to albumin. In addition to regulating chronobiological functions and the sleep-wake cycle, melatonin exhibits antioxidant [2], immunomodulatory, and anti-inflammatory effects, contributing to cellular membrane integrity [4].

Studies have demonstrated the performance-enhancing effects of immediate melatonin intake during wakefulness on endurance exercise until exhaustion at an intensity corresponding to the maximum aerobic capacity (iMAC) and energy metabolism [5–8]. Furthermore, melatonin promotes fat oxidation by enhancing energy expenditure and stimulating lipolysis, as evidenced in mice fed a high-fat diet where melatonin supplementation led to a reduction in adiposity and improved lipid utilization [9]. Additionally, melatonin preserves glycogen and accelerates post-exercise glycogen replenishment [7, 8]. The effects of melatonin on post-exercise carbohydrate and lipid metabolism, including its regulatory influence on the expression of GLUT4 and FAT/CD36, have been observed in rats [7]. The recovery process after physical exercise encompasses a complex interplay of physiological factors, including redox homeostasis, muscle tissue repair, inflammatory modulation, and energy metabolism [10], with a central role being played by the liver and skeletal muscle tissues [11].

Acylcarnitines, which are pivotal lipid intermediates, are implicated in intracellular signaling cascades, fatty acid transport, and metabolic regulation [12]. However, the precise quantitative impact of individual acylcarnitines on the total acycarnitine pool remains unknown. Their aberrant accumulation in hepatic and skeletal muscle tissues often accompanies chronic inflammatory states and metabolic dysregulation [13], indicating potential perturbations in mitochondrial metabolism [14]. Additionally, given their overlapping physiological roles in facilitating fatty acid transport into the mitochondria for β-oxidation, both melatonin and acylcarnitines may act synergistically to modulate lipid metabolism. Notably, reduced circulating levels of acylcarnitines have been linked to higher physical activity levels, enhanced cardiorespiratory fitness, and decreased adiposity, physiological adaptations that collectively reflect improved β-oxidative capacity [13]. Acylcarnitines are key intermediates in energy metabolism that support skeletal muscle during post-exercise recovery and may enhance lipid oxidation and energy production. Metabolomic analysis is a crucial method for assessing the acylcarnitine pool, allowing for the simultaneous identification and quantification of various metabolites, including acylcarnitines, in biological samples [15]. Moreover, metabolomics exhibits a remarkable ability to detect subtle alterations in the metabolic profile that may be undetectable using conventional analytical techniques [16].

However, previous investigations have predominantly focused on variable-duration exercises (i.e., time-to-exhaustion bouts), which inherently lead to inconsistencies in the total workload and metabolic stress. It is therefore necessary to investigate the effects of melatonin under more controlled exercise conditions to allow better standardization across animals. Moreover, most studies have assessed recovery only within the first few hours post-exercise, overlooking the later phases of metabolic adaptation. Extending the evaluation window to 24 h post-exercise is crucial to capture the delayed or sustained metabolic effects of melatonin, which may not be evident in the immediate recovery period. While melatonin is known to modulate lipid metabolism, its specific effects on the acylcarnitine profile, which are key intermediates in fatty acid oxidation and mitochondrial function, remain poorly understood. The current study thus aims to elucidate the impact of melatonin on serum and tissue metabolic profiles during the recovery phase following endurance exercise. We hypothesized that melatonin would decrease the acylcarnitine pool, optimize muscle lipid utilization, and preserve glycogen stores.

## Materials and methods

### Animals and environmental conditions

Thirty male Wistar rats (*Rattus norvegicus albinus*) were housed from 45 days of age in polypropylene cages (40 cm length, 30 cm width, 15 cm height, with five animals per cage) with food and water provided *ad libitum*. Environmental conditions were maintained throughout the experiment, including temperature (22 ± 2 °C), relative humidity (45-55%), noise (< 85 decibels), and a 12-hour light/dark cycle, with lights off at 06:00 h (zeitgeber time 12). Incandescent lamps (565-590 nm; 60 lx, measured with a lux meter) were used during the 12-hour light cycle. Reflectors were covered with a red filter (ROSCO brand, model # fire19; >600 nm; <15 lx; [17] to conduct experimental interventions with the rats during the dark cycle. The experimental procedures were conducted in accordance with Ethical Principles in Animal Research and approved by the Animal Ethics Committee of an institution affiliated with one of the authors. All procedures followed the ARRIVE guidelines 2.0.

### Experimental design

**Fig. 1:**
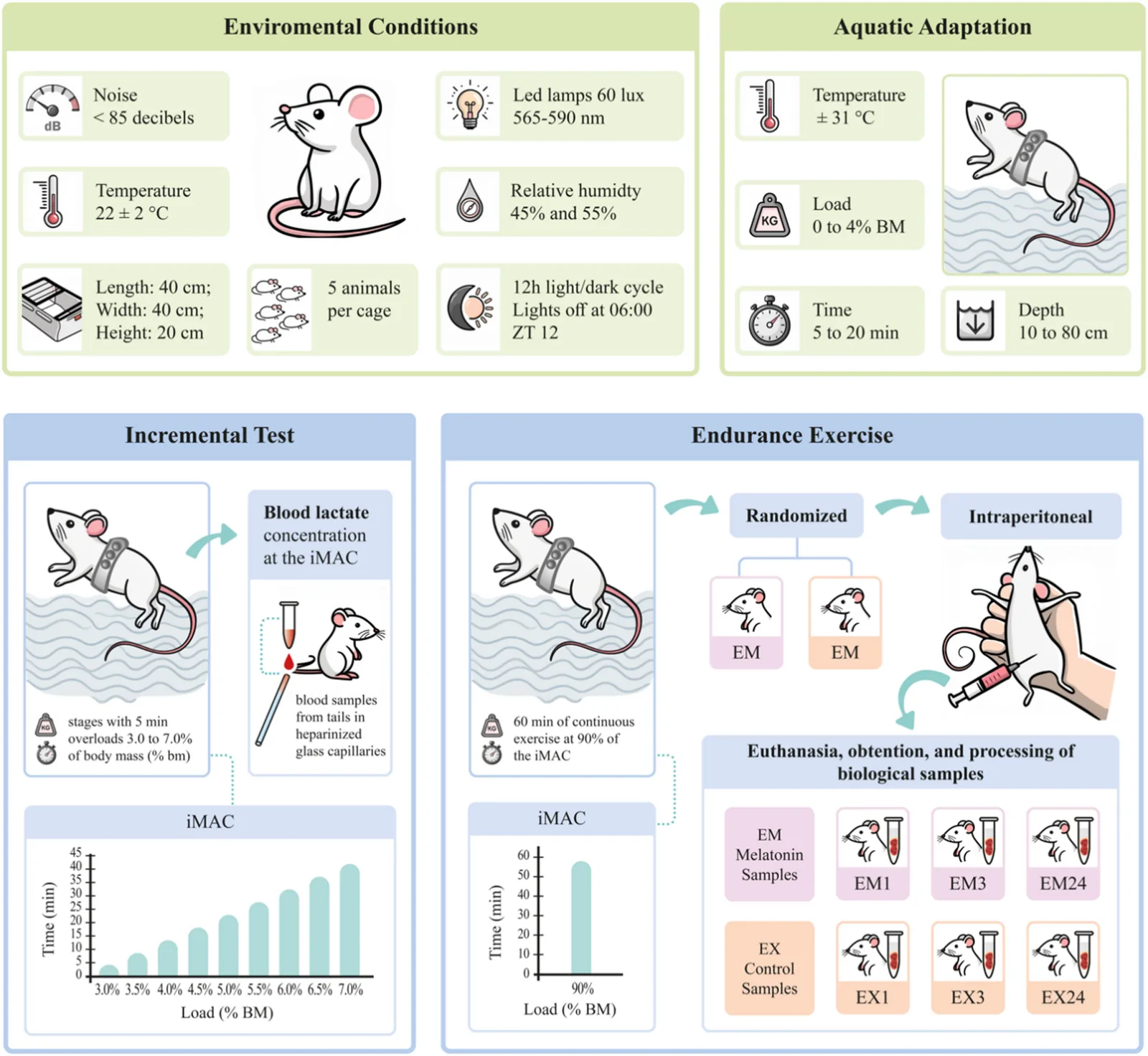
Experimental design of the study illustrating the steps from beginning to end of the experiment. ZT: zeitgeber time; iMAC: intensity of maximum aerobic capacity; BM: body mass; Ex1: Animals treated with vehicle solution and euthanized 1 h after exercise; Ex3: Animals treated with vehicle solution and euthanized 3 h after exercise; Ex24: Animals treated with vehicle solution and euthanized 24 h after exercise; EM1: Animals treated with melatonin and euthanized 1 h after exercise; EM3: Animals treated with melatonin and euthanized 3 h after exercise; EM24: Animals treated with melatonin and euthanized 24 h after exercise

After aquatic adaptation and the endurance swimming test, all animals performed the endurance swimming exercise. They were then randomly allocated into six groups (n = five per group), as follows: Exercised (E) and treated with melatonin (M, 10 mg.kg^-^¹), euthanized 1 h (EM1), 3 h (EM3), or 24 h (EM24) post-exercise; or Exercised and treated with vehicle solution (Ex, 0.9% NaCl and ethanol < 0.1%), euthanized 1 h (Ex1), 3 h (Ex3), or 24 h (Ex24) after the endurance swimming exercise (Fig. 1). No formal allocation concealment was applied during group assignment and data analyses were performed without blinding to group allocation. The sample size was calculated using GPower 3.1 software. No inclusion/exclusion criteria were established.

### Exercise protocol

At 80 to 89 days of age, the rats underwent a structured adaptation period to the aquatic environment and swimming protocol. They were exposed to water for 5 to 20 min per session, with progressively increasing depth (10-80 cm) and load (0 or 4% of body mass), in cylindrical, opaque tanks measuring 100 cm in height, 80 cm in water depth, and 30 cm in diameter. At 90 days of age, each animal performed an incremental test (IT) to determine the intensity corresponding to maximal aerobic capacity (iMAC). The IT involved progressive increases in load to identify the point at which a disproportionate rise in blood lactate concentration occurred, following the method described by Beck et al. [18]. The protocol consisted of 5-min stages with overloads ranging from 3.0 to 7.0% of body mass (% bm), increasing by 0.5% intervals, with the weights secured to the animals’ chests using elastic straps. After each stage, blood samples (25 µL) were collected from the distal portion of the tail to determine lactate concentration. The relationship between exercise intensity and blood lactate levels was plotted and the iMAC was determined by interpolation on the Y-axis. This test served to establish the intensity for the subsequent exercise session. At 100 days of age, all animals (body mass: 396.07 ± 4.40 g, with comparable body mass between the melatonin and vehicle groups, indicating appropriate standardization of the experimental conditions) performed a continuous 60-min swimming session at 90% of iMAC (zeitgeber time 12), the swimming endurance test, under the supervision of the same researcher (Fig. 1).

### Determination of blood lactate concentration

During the swimming endurance test, blood samples (25 µL) were collected from the animals’ tails using heparinized and calibrated glass capillaries. The samples were placed into plastic microtubes (1.5 mL) containing 400 µL of trichloroacetic acid (4%), mixed, and stored at 4 °C. After shaking and centrifugation (3000 rpm for 3 min), 50 µL of the supernatant was extracted and transferred to a 96-well microplate where 250 µL of reactive solution (glycine/EDTA and hydrazine hydrate stock), NAD (β-nicotinamide adenine dinucleotide), and LDH (L-lactic dehydrogenase, bovine heart), prepared for immediate use, were added. The samples were subjected to spectrophotometric measurements (Spectramax i3, Molecular Devices; San José, CA, USA) at 340 nm to compare sample values to a standard curve constructed from a serial dilution of 1-15 mmol.L^-^¹ of L-lactate [19].

### Melatonin administration

Melatonin (Sigma Aldrich Chemical Corporation; St. Louis, MO, USA; M-5250, > 98%) was dissolved in ethanol (< 0.1%) and diluted in saline (0.9% NaCl) for administration at a dose of 10 mg.kg^-^¹ [5]. The preparation was carried out just prior to use and was stored in an amber bottle wrapped in aluminum foil. Control animals received an equal volume of the vehicle alone (0.9% NaCl and ethanol < 0.1%). Administration was intraperitoneal and occurred immediately after the endurance swimming exercise.

### Euthanasia, obtention, and processing of biological samples

The animals were euthanized 1, 3, or 24 h after the end of the experimental procedures by decapitation, in accordance with the guidelines of the American Veterinary Medical Association [20]. After euthanasia, an aliquot of approximately 2.0 mL of blood was obtained and allowed to rest for 20 min (4 °C) before subsequent centrifugation (15 min, 3000 rpm, 10 °C). These serum samples were stored in different aliquots to avoid freeze-thaw cycles and were kept at -20 °C for further analysis. Then, skeletal muscle tissues (soleus, gluteus maximus, red and white portions of gastrocnemius) and liver were collected, immediately frozen in liquid nitrogen, and stored at -80 °C for further analyses.

### Blood glucose analysis

**Fig. 2:**
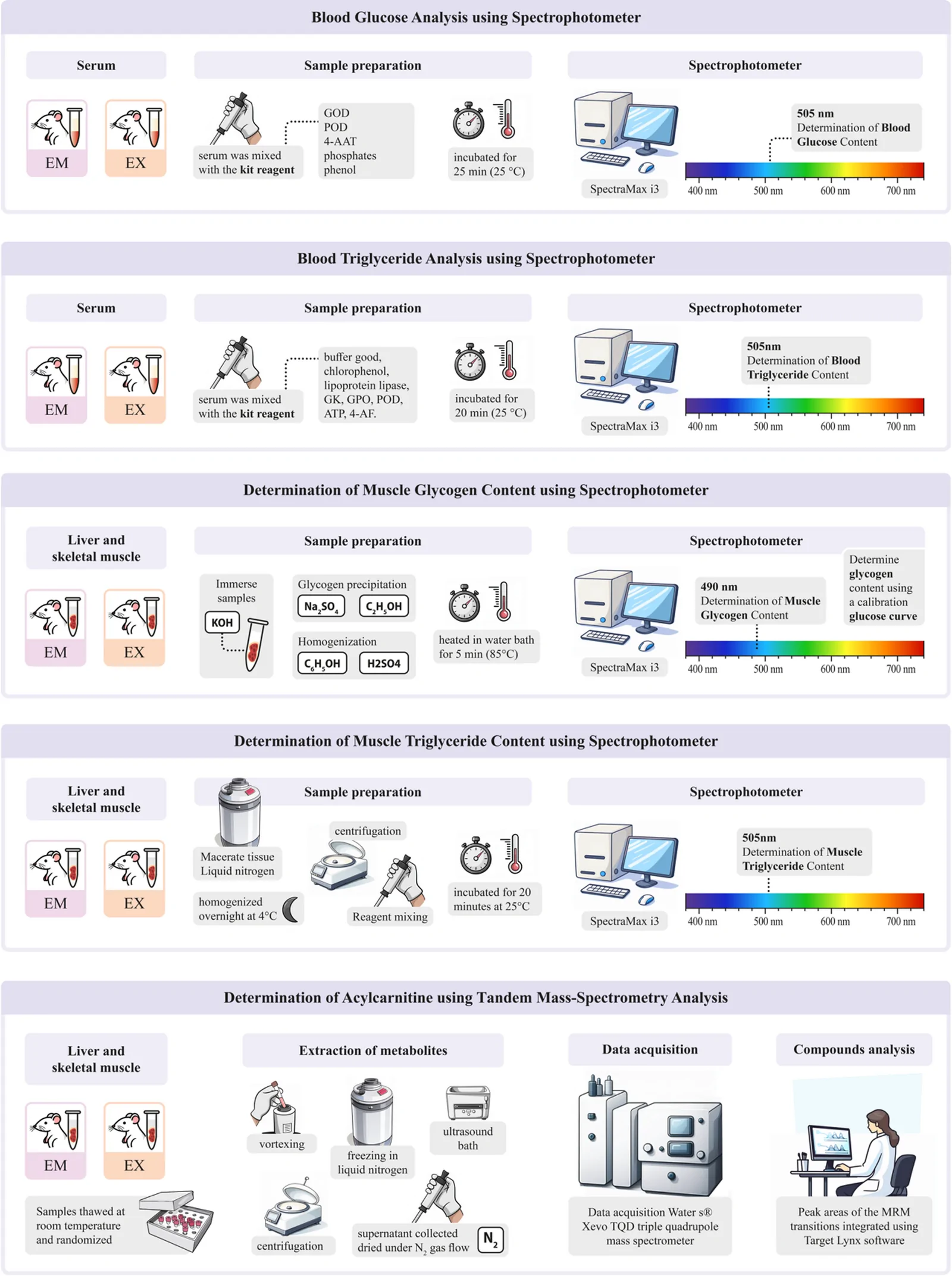
Schematization of the analysis methods of the present study. Blood glucose analysis using Spectrophotometer; Blood triglyceride analysis; Determination of muscle glycogen content; and Determination of muscle triglyceride content using spectrophotometer; Determination of acylcarnitine using tandem mass-spectrometry analysis. Analytical workflow for acylcarnitine profiling in muscle and liver tissues. The complete pipeline comprised four sequential stages, as follows: (i) Tissue extraction through mechanical (freeze-thaw cycles in liquid nitrogen, 1 min per cycle) and ultrasonic (40-45 kHz, 4 °C) disruption followed by centrifugation to obtain lipid-rich supernatants; (ii) Flow injection analysis (FIA) coupled to triple quadrupole mass spectrometry (Waters Xevo TQD; Shimadzu LC-20AD pump) enabling rapid sample throughput (4 min per injection) without compromising specificity; (iii) Multiple reaction monitoring (MRM) quantification of acylcarnitine species (C0-C18) via optimized precursor-to-product ion transitions terminating at the diagnostic carnitine head-group fragment (m/z 85.0); and (iv) Peak area integration and compound identification using TargetLynx software with reference to MRM parameters, detailed in Supplementary Table S1. Both endurance exercise Melatonin (EM) and Exercise control (Ex) groups underwent identical processing protocols to ensure analytical reproducibility and minimize bias

To determine blood glucose levels, 3 µL of serum was mixed with the kit reagent (300 µL; LaborLab; Guarulhos, SP, Brazil) and incubated for 25 min (25 °C). The kit reagent is composed of GOD (≥ 15 kU.L^-1^), POD (≥ 2 kU.L^-1^), 4-AAT (0.5 mmol.L^-1^), phosphates (pH = 7.5, 250 mmol.L^-1^), and phenol (5 mmol.L^-1^). The glucose absorbance was determined using a spectrophotometer (SpectraMax i3, Molecular Devices; San José, CA, USA) at 505 nm according to the kit’s guidelines. Blood samples were collected into standard serum tubes and immediately kept on ice (4 °C). To minimize glucose degradation, samples were allowed to rest for 20 min before centrifugation at 10 °C (3000 rpm, 15 min). After centrifugation, serum was aliquoted and stored at -20 °C until analysis, avoiding freeze-thaw cycles. Each group had five animals for the present analysis (Fig. 2).

### Blood triglyceride analysis

To determine blood triglyceride levels, 3 µL of serum was mixed with the kit reagent (300 µL; LaborLab; Guarulhos, SP, Brazil) and incubated for 20 min (25 °C). The kit reagent is composed of buffer (pH = 6.8, 50 mmol.L^-1^), chlorophenol (2 mmol.L^-1^), lipoprotein lipase (≥ 800 U.L^-1^), GK (≥ 500 U.L^- =1^), GPO (≥ 1500 U.L^-1^), POD (≥ 900 U.L^-1^), ATP (2 mmol.L^-1^), and 4-AF (0.4 mmol.L^-1^). The triglyceride absorbance was determined using a spectrophotometer (SpectraMax i3, Molecular Devices; San José, CA, USA) at 505 nm according to the kit’s guidelines. Each group had five animals for the present analysis (Fig. 2).

### Determination of muscle glycogen content

The glycogen content in skeletal muscle (gluteus maximus, red and white portions of the gastrocnemius) and the liver was determined as described by Dubois et al. [21]. Both skeletal muscle (250 mg) and liver (500 mg) were initially immersed in potassium hydroxide (30%; Êxodo Científica; Sumaré, SP, Brazil) and then mixed with a saturated sodium sulfate solution (20 µL; Dinâmica Química Contemporânea Ltda; Indaiatuba, SP, Brazil) and ethanol (70%) for glycogen precipitation. The samples were homogenized with phenol (10 µL; Êxodo Científica; Sumaré, SP, Brazil) and sulfuric acid (2 mL; Dinâmica Química Contemporânea Ltda; Indaiatuba, SP, Brazil), and heated in a water bath for 5 min (85 °C). Finally, the absorbance was measured using a spectrophotometer (Hach Company, Loveland, CO, USA; 490 nm), and the glycogen content was calculated using a calibration glucose curve. Each group had five animals for the present analysis (Fig. 2).

### Determination of muscle triglyceride content

To determine triglyceride content, liver and skeletal muscle samples (soleus, gluteus maximus, red and white portions of the gastrocnemius) (100-200 mg) were macerated using liquid nitrogen and then transferred to plastic tubes containing Triton X-100 (1%) at the ratio of 200 mg of tissue per 1 mL of Triton. Subsequently, the samples were homogenized overnight at 4 °C using magnetic stir bars (5 × 3 mm). After homogenization, the samples were centrifuged at 4000 rpm for 10 min, and 10 µL of the supernatant was pipetted into a 96-well microplate and mixed with the kit reagent (200 µL; LaborLab; Guarulhos, SP, Brazil). The mixture was then incubated for 20 min at 25 °C. Triglyceride absorbance was measured using a spectrophotometer (SpectraMax i3, Molecular Devices; San José, CA, USA) at 505 nm, according to the kit’s guidelines. Each group had five animals for the present analysis (Fig. 2).

### **Acylcarnitine extraction and quantification**

Acylcarnitine determination was conducted using flow injection analysis coupled to tandem mass spectrometry (FIA-MS/MS), following the established methodology of Sarafian et al. [23], with tissue-specific adaptations previously validated in our laboratory [22]. The complete analytical workflow, from tissue extraction through data acquisition and quantification, is schematically represented in Fig. 2. Tissue samples (50-150 mg) were thawed at room temperature and extracted with isopropanol pre-cooled to 4 °C in a ratio of 1:10 (mg tissue:µL solvent). This ratio proved essential for efficient protein precipitation while retaining lipid intermediates intact. After initial vortexing (30 s), samples underwent three freeze-thaw cycles in liquid nitrogen (1 min intervals), each followed by thorough vortexing to ensure complete cell membrane disruption in muscle and hepatic tissues. Sonication was performed for 10 min at 4 °C (40-45 kHz ultrasonic frequency), followed by a final 30 s vortex. After centrifugation at 12,000 rpm for 10 min at 4 °C, the supernatant (400 µL) was carefully transferred to a clean tube, avoiding the protein pellet, and evaporated to dryness under a gentle stream of nitrogen gas. The dried extract was reconstituted in 250 µL of mobile phase solution (acetonitrile: water, 82:18 v/v, acidified with formic acid 0.1%) containing the internal standard L-Leucine-5,5,5-d₃ at 0.73 µg.mL^-^¹ to correct for potential ionization suppression artifacts.

Data acquisition was conducted on a Waters Xevo TQD triple quadrupole mass spectrometer interfaced with a Shimadzu LC-20AD pump system, utilizing procedures adapted from Moura [24]. Rather than employing liquid chromatography with a packed column, flow injection analysis (FIA) was selected to enable rapid analysis (4 min per sample) without compromising specificity. The mobile phase flow was programmed as a gradient from 0.01 to 0.50 mL.min^-^¹. Positive electrospray ionization (ESI+) was used to generate molecular ions. Acylcarnitine species (C0-C18) were quantified via multiple reaction monitoring (MRM), with each analyte monitored using precursor-to-product ion transitions specific to the carnitine moiety (m/z 85.0), as detailed in Supplementary Table S1. As depicted in Fig. 2, this analytical pipeline integrated four critical stages, as follows: metabolite extraction under controlled temperature conditions, high-throughput FIA-MS/MS data acquisition, MRM-based quantification targeting the diagnostic carnitine fragment, and compound identification via TargetLynx software. Quantification was performed by measuring the peak area ratio of analyte to internal standard and referencing this value against a 7-point calibration curve spanning 0.1-100 µmol.L^-^¹ (R² consistently > 0.99). Tissue concentrations were normalized to wet tissue weight and reported in µmol.g^-^¹. Quality control (QC) samples were prepared from pooled tissue extracts and interspersed throughout each analytical batch (every 10 injections) to monitor instrumental performance. Results were accepted only when QC values fell within ± 15% of the expected concentration, ensuring data integrity throughout the run.

### Statistical analysis

The data are presented as mean ± standard error of the mean. Normality was verified using the Shapiro-Wilk test (*p* > 0.05). Data normality was assessed using the Shapiro-Wilk test. Variables that did not meet normality assumptions were transformed using an inverse (1/x) transformation, after which parametric statistical analyses were applied. A two-way ANOVA followed by a Newman-Keuls post hoc test was applied to analyze the effects of melatonin (two levels: with or without melatonin) and the effect of time (three levels: 1, 3, and 24 h). Effect size analysis (E.S) [25] was used as a complementary test. The thresholds for small, moderate, and large effects were 0.20, 0.50, and 0.80, respectively. All large effect sizes were included in the figures. A significance level of 5% and Statistica 7.0 (StatSoft, Inc.; Tulsa, OK, USA) were used for all analyses. In addition, the Integrated Biomarkers Responses (IBR) was used according to the instructions of Beliaeff & Burgeot [26] to observe all amino acids in a single analysis, through the area calculation of a star plot for each acylcarnitine, generating the IBR-value. The vehicle group was always the control of the melatonin group within each time point. There was no exclusion of data in the present analyses. Given the number of dependent variables analyzed, statistical tests were conducted in an exploratory manner and p-values were not adjusted for multiple comparisons.

## Results

The iMAC intensity was 48.25 ± 2.7 min, 5.1 ± 0.4% BM, and 4.7 ± 0.6 mmol.L^-^¹, and all animals completed the 60 min of the swimming endurance exercise.

**Fig. 3:**
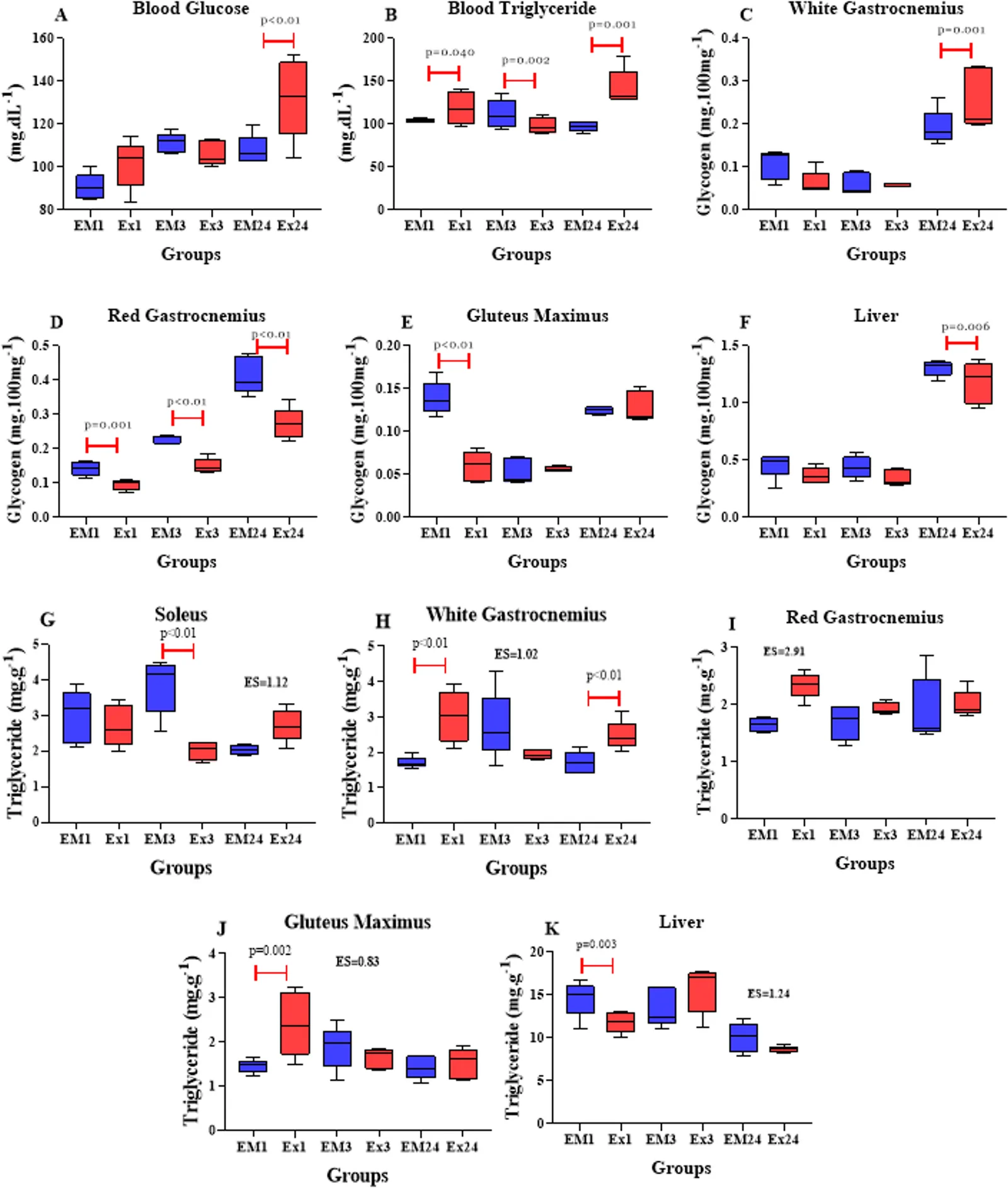
Metabolic results of serum and tissue substrates at different moments. The graphs represent the means and standard errors for serum parameters. (**A**) Blood glucose and (**B**) blood triglyceride. Glycogen content in the white (**C**) and red (**D**) gastrocnemius portion, gluteus maximus (**D**), and liver (**F**). Triglyceride content in the soleus (**G**), and the white (**H**) and red (**I**) gastrocnemius portion, gluteus maximus (**J**), and liver (**K**) at different moments. 1 h: animals that were euthanized 1 h after endurance exercise. 3 h: animals that were euthanized 3 h after endurance exercise. 24 h: animals that were euthanized 24 h after endurance exercise. Red bars represent *p* < 0.05 between groups for the same time, E.S: effect size

Serum parameters outcomes are shown in Fig. 3A and B. Melatonin caused a reduction in glucose levels (F = 11.01; *p* < 0.001), while ANOVA showed no effect for triglyceride levels (F = 1.08; *p* > 0.005). A notable effect of time was observed, with a marked increase in glucose levels especially for Ex24 (F = 12.34; *p* < 0.001).

The glycogen data are displayed in Fig. 3C, D, E and F. Melatonin exhibited a significant increase in the red gastrocnemius (F = 82.81; *p* < 0.001), gluteus maximus (F = 19.55; *p* < 0.001), and liver (F = 6.24; *p* < 0.005). Conversely, it resulted in a reduction in the white gastrocnemius (F = 4.28; *p* < 0.005). There was a notable effect of time on glycogen content, with an increase observed in the white gastrocnemius (F = 78.96; *p* < 0.001), red gastrocnemius (F = 215.38; *p* < 0.001), gluteus maximus (F = 88.80; *p* < 0.001), and liver (F = 322.69; *p* < 0.001).

The tissue triglyceride data are displayed in Fig. 3G, H, I, J and K. Melatonin elicited a significant reduction in the white gastrocnemius (F = 20.79; *p* < 0.01), red gastrocnemius (F = 8.76; *p* < 0.005), and gluteus maximus (F = 4.90; *p* < 0.005) while demonstrating an increase in the soleus (F = 6.00; *p* < 0.005), with no effect on the liver (F = 3.44; *p* > 0.005). Moreover, a significant time effect on tissue triglyceride content was evident, with an increase observed in the red gastrocnemius (F = 3.96; *p* < 0.005) and a decrease in the soleus (F = 6.41; *p* < 0.005), gluteus maximus (F = 4.21; *p* < 0.005), and liver (F = 34.75; *p* < 0.001).

**Fig. 4:**
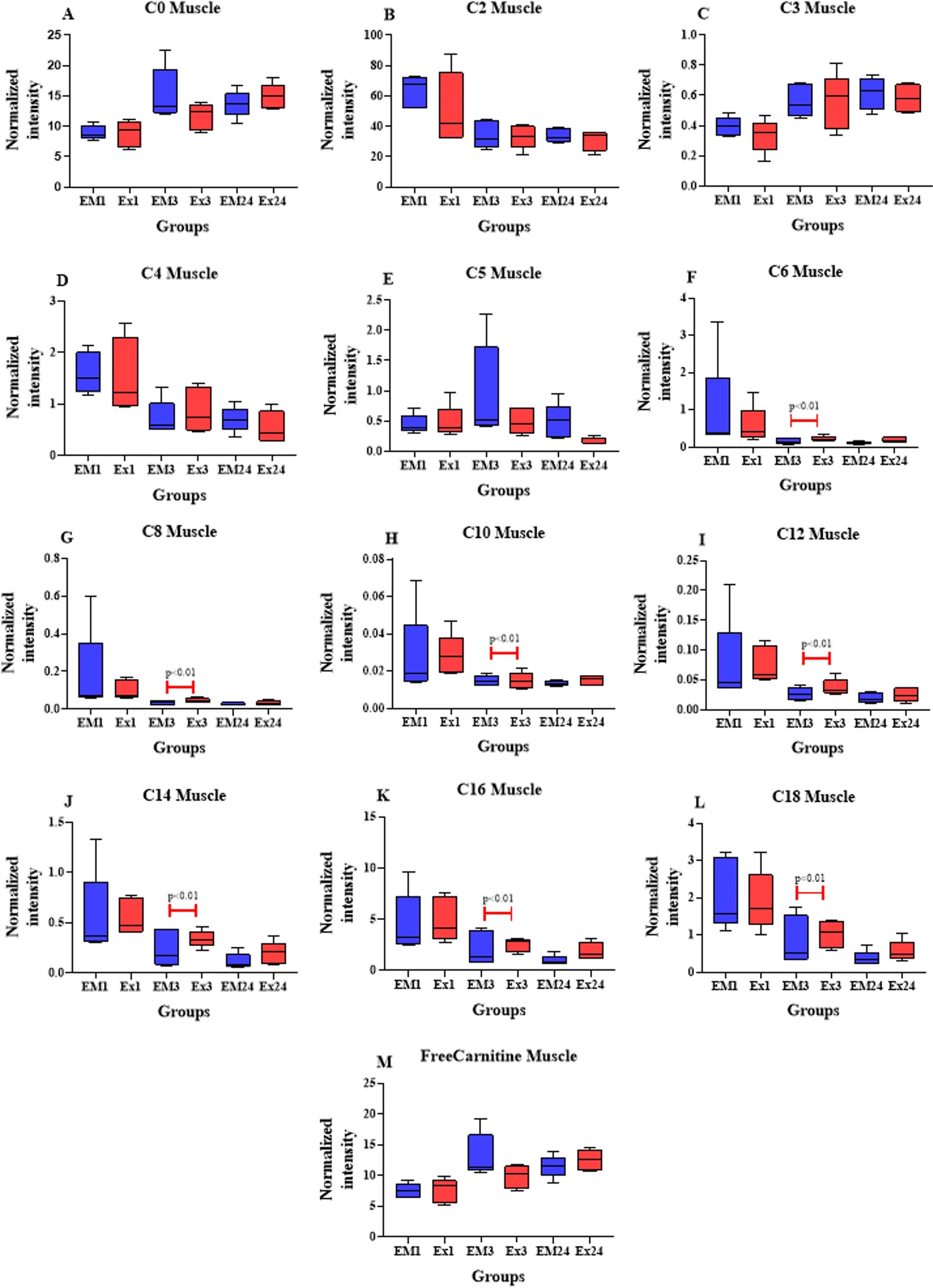
Acylcarnitine pool in gluteus maximus. The graphs represent the means and standard errors for carnitine content of C0 (**A**), C2 (**B**), C3 (**C**), C4 (**D**), C5 (**E**), C6 (**F**), C8 (**G**), C10 (**H**), C12 (**I**), C14 (**J**), C16 (**K**), C18 (**L**) and free carnitine (**M**) at different moments. 1 h: animals that were euthanized 1 h after endurance exercise. 3 h: animals that were euthanized 3 h after endurance exercise. 24 h: animals that were euthanized 24 h after endurance exercise. E.S: effect size

Regarding the gluteus maximus acylcarnitine pool, the data are displayed in Fig. 4. Melatonin administration yielded no significant impact. However, time (as a main effect) led to a decrease in several acylcarnitines, including C2 (F = 13.66, *p* < 0.001), C4 (F = 13.49, *p* < 0.001), C8 (F = 3.97, *p* < 0.005), C10 (F = 5.11, *p* < 0.005), C12 (F = 6.73, *p* < 0.001), C14 (F = 9.21, *p* < 0.001), C16 (F = 10.43, *p* < 0.001), and C18 (F = 16.16, *p* < 0.001). Conversely, free carnitine (F = 12.84, *p* < 0.001), C0 (F = 13.05, *p* < 0.001), and C3 (F = 11.70, *p* < 0.001) showed an increase over time.

**Fig. 5:**
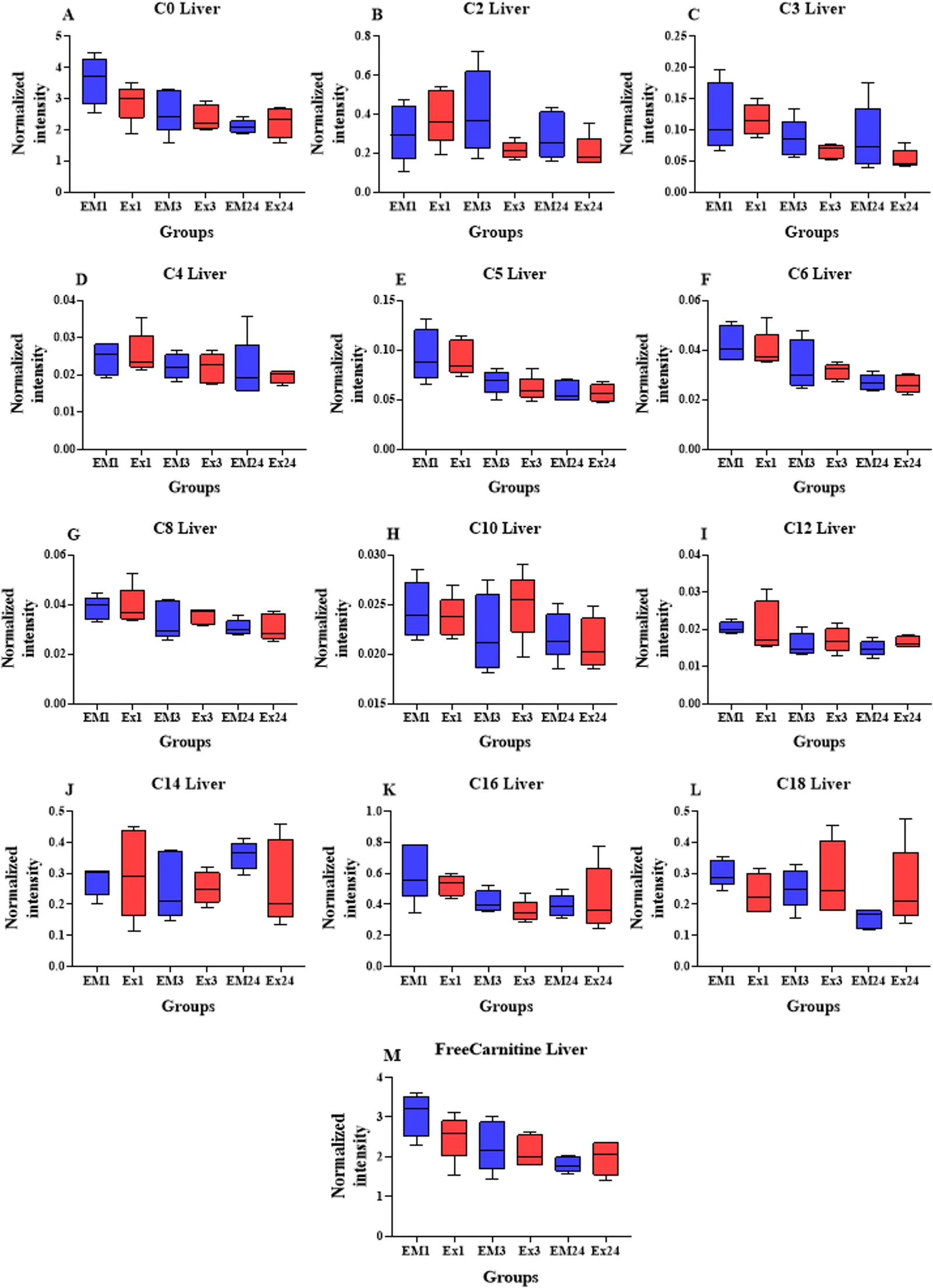
Acylcarnitine pool in liver. The graphs represent the means and standard errors for carnitine content of C0 (**A**), C2 (**B**), C3 (**C**), C4 (**D**), C5 (**E**), C6 (**F**), C8 (**G**), C10 (**H**), C12 (**I**), C14 (**J**), C16 (**K**), C18 (**L**) and free carnitine (**M**) at different moments. 1 h: animals that were euthanized 1 h after endurance exercise. 3 h: animals that were euthanized 3 h after endurance exercise. 24 h: animals that were euthanized 24 h after endurance exercise. E.S: effect size

The hepatic acylcarnitine pool is displayed in Fig. 5. Although melatonin had no effect, time led to a reduction in free carnitine (F = 8.77, *p* < 0.001), C0 (F = 9.60, *p* < 0.001), C3 (F = 5.48, *p* < 0.005), C5 (F = 14.49, *p* < 0.001), C6 (F = 13.61, *p* < 0.001), C8 (F = 5.52, *p* < 0.005), C12 (F = 5.19, *p* < 0.005), and C16 (F = 5.67, *p* < 0.005).

**Fig. 6:**
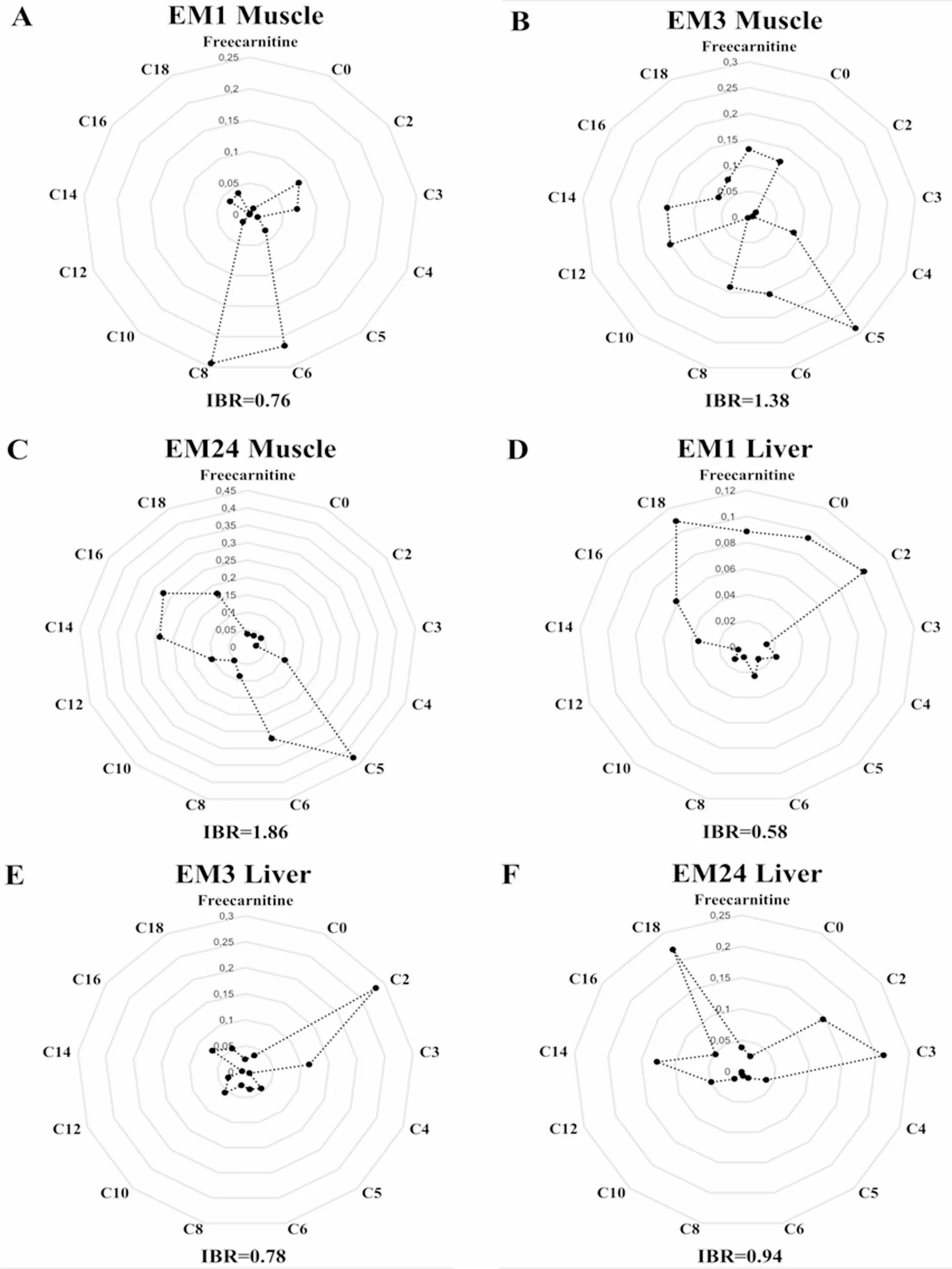
Integrated biomarker response (IBR) index. **(A)** EM1: Gluteus maximus of animals treated with melatonin and euthanized 1 h after exercise. **(B)** EM3: Gluteus maximus of animals treated with melatonin and euthanized 3 h after exercise. **(C)** EM24: Gluteus maximus of animals treated with melatonin and euthanized 24 h after exercise. **(D)** EM1: Liver of animals treated with melatonin and euthanized 1 h after exercise. **(E)** EM3: Liver of animals treated with melatonin and euthanized 3 h after exercise. **(F)** EM24: Liver of animals treated with melatonin and euthanized 24 h after exercise. The vehicle group is always the control of the melatonin group within each time point

The IBR results are shown in Fig. 6, demonstrating that melatonin caused more variation for C8 and C6 in muscle 1 h after the exercise, but with C5 being more impacted 3 and 24 h after the exercise. At the liver, while C18 was more influenced by melatonin 1 and 24 h after the exercise, C2 was more modified 3 h after the exercise.

## Discussion

Precise control over volume and intensity is crucial in experiments assessing the impact of physical exercise [27]. In the present study, we utilized fixed-duration exercise with individually determined intensity, which all animals successfully completed, highlighting the robustness and accuracy of the methods. It is important to clarify that this study did not aim to investigate the effects of exercise itself but rather to evaluate the specific influence of melatonin administration in trained rats, thereby isolating its role in metabolic recovery. Despite the absence of a decrease in the intramuscular acylcarnitine pool, melatonin induced notable changes in metabolic parameters, partially supporting our hypothesis.

This is, to the best of our knowledge, the first study to analyze the acute effect of melatonin administration following exercise on energy metabolism in rats subjected to an individualized fixed-duration exercise session at an intensity corresponding to their maximum aerobic capacity. While previous studies have explored the effects of melatonin on metabolism and oxidative stress, comprehensive investigations into its influence on acylcarnitine levels remain limited [28, 29].

Acylcarnitines play a crucial role in transporting fatty acids into the mitochondria for beta-oxidation, a process vital for cellular energy production. Free carnitine (C0) corresponds to the non-esterified form of carnitine and is frequently used interchangeably with C0 in metabolomic and clinical studies. Nevertheless, both parameters are presented separately in the present study to allow a more comprehensive evaluation of potential tissue-specific variations and subtle metabolic differences, as suggested in previous reports [30]. Long-chain fatty acids are activated in the cytosol as acyl-CoA but cannot directly cross the inner mitochondrial membrane [31]. To facilitate this transport, carnitine palmitoyltransferase I (CPT1) converts acyl-CoA into acylcarnitine at the outer mitochondrial membrane [32]. The acylcarnitine is then transported into the mitochondria via a specific transporter protein, where carnitine palmitoyltransferase II (CPT2) converts it back into acyl-CoA for beta-oxidation [33]. This mechanism is critical for generating ATP and supporting cellular energy metabolism. Specifically, long-chain acylcarnitines (C12-C18) were monitored in muscle and liver tissues as they are directly involved in the transport of long-chain fatty acids into mitochondria, and their modulation may reflect subtle adjustments in lipid utilization and energy metabolism in response to melatonin administration [34]. Melatonin has been implicated in the regulation of fatty acid metabolism, particularly by enhancing fatty acid transport into the mitochondria for beta-oxidation [35]. This process is crucial during periods of increased metabolic demand, such as during exercise recovery. Melatonin enhances the activity of CPT1, a key enzyme responsible for long-chain fatty acid transport across the mitochondrial membrane [36]. By promoting this transport, melatonin may improve beta-oxidation efficiency, supporting energy homeostasis and reducing the accumulation of fatty acid intermediates in cells.

Given the physiological similarities in facilitating fatty acid transport for beta-oxidation, we hypothesized that melatonin would increase fat oxidation and reduce the acylcarnitine pool in both muscle (gluteus maximus) and liver tissues. However, this was not observed, as the acylcarnitine pool appeared unresponsive to the effects of melatonin in the context of exercise. This lack of convergence between melatonin and the acylcarnitine pool suggests that enhanced lipid utilization in melatonin-treated groups may be due to increased expression of FAT/CD36 [7]. Melatonin effectively reduced the tissue triglyceride content in various skeletal muscle groups, consistent with previous studies [8]. These authors attributed this reduction to the increased expression of FAT CD36, a key protein involved in lipid metabolism. It is important to note that, unlike Faria et al. [7], who administered melatonin prior to exercise to investigate its effects on exercise performance, in the present study, melatonin was administered immediately after the endurance swimming session. This post-exercise intervention was intended to assess the impact of melatonin on recovery and tissue bioenergetics, including modulation of acylcarnitine profiles, triglyceride content, and lipid utilization pathways such as FAT/CD36. This difference in timing may help explain variations in metabolic outcomes observed between studies. Mendes et al. [37] examined treadmill training (20 m.min^-1^, 5 days.week^-1^, 16 weeks) and treatment with melatonin (10 mg.kg.day^-1^, 8 weeks) in Wistar rats, observing enhanced energy metabolism efficiency, characterized by decreased body weight and heightened insulin sensitivity. However, it is crucial to acknowledge that the exercise regimen in our study was acute. This illustrates the role of melatonin in reducing serum triglyceride levels, likely by enhancing the transfer of triglycerides from circulation to skeletal muscles for oxidation. As previously mentioned, melatonin may influence FAT/CD36 expression, which could potentially reduce dependence on acylcarnitines, possibly due to the preferential facilitation of the transport of larger fatty acid molecules. The latter interpretation may help explain the findings of the present study. Nevertheless, this remains a speculative hypothesis and further studies are required to substantiate this possibility. A recent study demonstrated the effect of melatonin in reducing the acylcarnitine pool in breast cancer-bearing (BC) mice; however, the mechanisms responsible for this reduction are not well understood [38]. Moreover, in muscle, although conventional statistical analyses did not produce significant results, the IBR demonstrated that melatonin caused more variation for C8 and C6 in muscle 1 h after the exercise, but with C5 being more impacted 3 and 24 h after the exercise. In the liver, while C18 was more influenced by melatonin 1 and 24 h after exercise, C2 was more modified 3 h after exercise (Fig. 6). These results highlight that the time of analysis after exercise can impact different acylcarnitine levels, warranting future studies to fully understand their specific relevance in the recovery process.

Melatonin caused a notable reduction in glucose levels 24 h post-exercise (*p* < 0.001). The role of melatonin in glucose homeostasis is well documented as it improves glucose tolerance and insulin sensitivity [39], leading to decreased circulating glucose levels, as observed in the current study. The reduction in glucose suggests an increased utilization of glucose by tissues, contributing to enhanced replenishment of glycogen stores [40]. A significant increase in muscle glucose uptake is essential to meet energy demands during post-endurance exercise recovery [41]. This uptake is facilitated by the translocation of the glucose transporter GLUT4 to the sarcolemma and transverse tubules, enabling diffusion [42]. Although we did not assess GLUT4 expression in this study, the findings of Faria et al. [8] indicate that in melatonin-treated and exercise-exposed rats, increased GLUT4 expression was associated with enhanced glycogen replenishment in tissues. This likely mirrors the elevated glycogen stores observed in the present study.

Glycogen is essential for exercise performance, particularly during high-intensity and prolonged exercise [43]. Research indicates that glycogen levels are depleted following swimming exercises in rats, especially in muscles such as the gluteus maximus [44]. Rapid glycogen replenishment is crucial for preparing muscles for subsequent physical activities [41]. In this study, melatonin administration resulted in significant increases in glycogen content in the red gastrocnemius (*p* < 0.001), gluteus maximus (*p* < 0.001), and liver (*p* < 0.005) compared to the control groups. These findings suggest that melatonin may accelerate the restoration of energy substrates, thereby enhancing glycogen reserves, which is consistent with the observations of Mazepa et al. and Faria et al. [45, 7]. Furthermore, our results align with those of Mendes et al. [40], who reported upregulation of PI3K, GLUT4, and glycogen storage in the skeletal muscles of rats subjected to treadmill training. Interestingly, the impact of melatonin appears to be tissue-dependent, as the white gastrocnemius displayed a reduction in glycogen content 24 h post-exercise in the melatonin group. The graphic abstract summarizing the main findings is provided in the Supplementary Material.

Although different intensities and durations produce different physiological scenarios, our study was concerned with correctly individualizing the exercise loads for each animal. Furthermore, caution is needed when making inferences to the human model, where melatonin generally does not show such an ergogenic effect [46] and the sleep-wake cycle is different, with rats being active during the night.

## Conclusion

In conclusion, our study demonstrates that melatonin and acylcarnitines do not appear to act synergistically in modulating β-oxidation following endurance swimming exercise, as the acylcarnitine pool remained insensitive to melatonin administration in the trained rats. However, melatonin significantly affects other metabolic parameters, including glucose regulation, glycogen preservation, and tissue triglyceride content. These results highlight the potential of melatonin to enhance post-exercise metabolic recovery, particularly through mechanisms involving carbohydrate and lipid metabolism. Furthermore, the data suggest a possible preferential role of melatonin in facilitating the transport of larger fatty acid molecules, which may contribute to its bioenergetic benefits during recovery. Future studies assessing temporal metabolic dynamics are essential to fully elucidate the role of melatonin in exercise recovery.

## Electronic Supplementary Material

Below is the link to the electronic supplementary material.

Supplementary Table S1 (DOCX 7.82 KB)

## Data Availability

The data that support the findings of this study are available from the corresponding authors upon reasonable request.
