# Supplementary material for: Effects of melatonin on muscle and liver bioenergetic profiles: focus on acylcarnitines following endurance swimming exercise in rats
Source: Hormones (Athens). 2026 Jun 8;25(2):489–503. doi: 10.1007/s42000-026-00796-x (PMC13582139; doi:10.1007/s42000-026-00796-x)
Supplement: Supplementary file 1 [file 42000_2026_796_MOESM1_ESM.docx]

Supplementary Material

**Supplementary Table S1**: Acylcarnitines investigated and their experimental detection parameters.

| **Compound** | **Cone (V)** | **Collision (eV)** | **MRM (Transition, *m/z*)** |
| --- | --- | --- | --- |
| Free Carnitine (C0)  Acetyl-Carnitine (C2)  Propionyl-Carnitine (C3)  Butyryl-CarnitineButyryl - Carnitine (C4)  Isovaleryl-Carnitine (C5)  Hexanoyl-Carnitine (C6)  Octanoyl-Carnitine (C8)  Decanoyl-Carnitine (C10)  Tetradecanoyl - Carnitine (C14)  Palmitoyl-Carnitine (C16)  Hydroxypalmitoyl-Carnitine (C16OH)  Octadecanoyl-Carnitine (C18)  Octadecenoyl-Carnitine (C18:1)  Malonyl-Carnitine (C3DC) | 40  35  35  35  35  35  40  40  40  40  40  40  40  40 | 19  19  19  19  19  19  19  19  28  25  25  19  19  19 | 162.0-85.0  204.1-85.0  218.1-85.0  232.1-85.0  246.1-85.0  260.0-85.0  288.1-85.0  316.0-85.0  372.0-85.0  400.3-85.0  416.0-85.0  428.0-85.0  426.0-85.0  248.0-85.0 |
